# Nonalcoholic Fatty Liver Disease: Focus on New Biomarkers and Lifestyle Interventions

**DOI:** 10.3390/ijms22083899

**Published:** 2021-04-09

**Authors:** Maria Notarnicola, Alberto Ruben Osella, Maria Gabriella Caruso, Pasqua Letizia Pesole, Antonio Lippolis, Valeria Tutino, Caterina Bonfiglio, Valentina De Nunzio, Maria Principia Scavo, Antonella Mirizzi, Isabella Franco, Tamara Lippolis, Rosalba D’Alessandro, Maria Grazia Refolo, Caterina Messa

**Affiliations:** 1 Laboratory of Nutritional Biochemistry, National Institute of Gastroenterology “S. de Bellis” Research Hospital, 70013 Castellana Grotte, BA, Italy; valeria.tutino@irccsdebellis.it (V.T.); valentinadx@hotmail.it (V.D.N.); lippolis.tamara@gmail.com (T.L.); 2 Laboratory of Epidemiology and Biostatistics, National Institute of Gastroenterology “S. de Bellis” Research Hospital, 70013 Castellana Grotte, BA, Italy; ar.osella@irccsdebellis.it (A.R.O.); catia.bonfiglio@irccsdebellis.it (C.B.); antonella.mirizzi@irccsdebellis.it (A.M.); isabella.franco@irccsdebellis.it (I.F.); 3 Ambulatory of Clinical Nutrition, National Institute of Gastroenterology “S. de Bellis” Research Hospital, 70013 Castellana Grotte, BA, Italy; gabriella.caruso@irccsdebellis.it; 4 Laboratory of Clinical Pathology, National Institute of Gastroenterology “S. de Bellis” Research Hospital, 70013 Castellana Grotte, BA, Italy; pesoleletizia@gmail.com (P.L.P.); antonio.lippolis@irccsdebellis.it (A.L.); 5 Laboratory of Personalized Medicine, National Institute of Gastroenterology “S. de Bellis” Research Hospital, 70013 Castellana Grotte, BA, Italy; maria.scavo@irccsdebellis.it; 6 Laboratory of Cellular and Molecular Biology, National Institute of Gastroenterology “S. de Bellis” Research Hospital, 70013 Castellana Grotte, BA, Italy; rosalba.dalessandro@irccsdebellis.it (R.D.); maria.refolo@irccsdebellis.it (M.G.R.); caterina.messa@irccsdebellis.it (C.M.)

**Keywords:** NAFLD, fatty acids, exosomes, cytokeratins, lipid metabolism, Mediterranean diet, physical activity

## Abstract

Nonalcoholic fatty liver disease (NAFLD) is considered a hepatic manifestation of metabolic syndrome, characterized from pathological changes in lipid and carbohydrate metabolism. Its main characteristics are excessive lipid accumulation and oxidative stress, which create a lipotoxic environment in hepatocytes leading to liver injury. Recently, many studies have focused on the identification of the genetic and epigenetic modifications that also contribute to NAFLD pathogenesis and their prognostic implications. The present review is aimed to discuss on cellular and metabolic alterations associated with NAFLD, which can be helpful to identify new noninvasive biomarkers. The identification of accumulated lipids in the cell membranes, as well as circulating cytokeratins and exosomes, provides new insights in understanding of NAFLD. This review also suggests that lifestyle modifications remain the main prevention and/or treatment for NAFLD.

## 1. Introduction

Nonalcoholic fatty liver disease (NAFLD) is considered the most common form of chronic liver disease worldwide [1,2]. It has been closely associated with metabolic syndrome, and its incidence is growing rapidly [1,2,3,4]. Furthermore, the presence of NAFLD has been reported in obese children and adolescents [5], that are certainly at higher risk of cardiovascular diseases and metabolic complications in adult age [6]. Epidemiologic studies have demonstrated that the excessive consumption of free sugars in childhood is associated with NAFLD in overweight adults [7,8]. 

Literature data indicate that the pathogenesis of NAFLD is closely linked with increased adiposity, dyslipidemia, and insulin resistance [9]. Recently, the influence of genetic factors in the development of hepatic steatosis, as well as its progression in more severe forms of liver disease, has been reported in both experimental and observational studies [10,11]. Genome wide association studies (GWAS) have demonstrated the presence of genetic variants associated with NAFLD [12]. The single nucleotide polymorphisms E167K of the Transmembrane 6 Superfamily Member 2 (TM6SF2) gene, and the polymorphisms I148M of the Patin-like Phospholipase-3 (PNPLA3) gene, are two common genetic variations conferring susceptibility to NAFLD [13,14]. The substitution of lysine for glutamate at residue 167 of TM6SF2 gene is associated with the loss of TM6SF2 function protein [15], causing a reduced hepatic lipid secretion via Very Low Density Lipoprotein (VLDL) and an increased fat accumulation in hepatocellular droplets [16]. PNPLA3- I148M variant is strongly associated with the severity of liver disease and, likely, it is related to the progression of steatosis and progressive fibrosis, up to hepatocellular carcinoma (HCC) [17].

Among key genes considered etiological drivers of NAFLD, the membrane bound *O*-acyltransferase domain containing 7 (MBOAT7) gene has been also identified as a genetic modifier of risk for liver damage progression [18]. One of its variants, namely rs641738 C > T polymorphism, has been shown to be associated with hepatic steatosis and severity of NAFLD-related necroinflammation [19]. 

In addition to the genetic component, emerging evidence suggests that epigenetic modifications may also contribute to the pathophysiology of NAFLD. Epigenetics refers to the reversible changes in gene expression caused by environmental stimuli [20]. In response to these external factors, as lifestyle habits and nutritional factors, the epigenetic modulation refers to DNA methylation, histone modifications, and the regulation of specific noncoding RNAs. Many studies have investigated the role of noncoding RNAs in the pathogenesis of NAFLD and their potential as biomarkers of the disease [21,22]. 

Noncoding RNAs are a group of RNA molecules without protein-coding capacity, involved in chromatin remodeling and in transcriptional and post-transcriptional gene regulation [23]. Among noncoding RNAs, especially the microRNAs (miRNA) are deregulated in NAFLD, and several studies have shown the association of miRNAs with liver steatosis [24,25,26].

Given the plethora of risk factors, the NAFLD has been considered a complex disease, not only restricted to the liver, but it is also often associated with other pathologies such as diabetes, cardiovascular, and chronic kidney diseases [27,28]. Several studies have demonstrated that NAFLD is a hepatic manifestation of metabolic syndrome, characterized by pathological changes in lipid and carbohydrate metabolism [1,29,30].

In the liver, fat accumulation seems to cause lipid peroxidation, producing into the hepatocyte high levels of Reactive Oxygen Species (ROS). Increased lipid peroxidation has been documented in animal models, as in the ob/ob mice where an increase in mitochondrial ROS production has been detected [31]. Abnormal peroxidation occurs both in the liver cell and circulating lipids causing lipotoxicity with metabolic dysfunctions [32,33].

The imbalance between lipid uptake/lipogenesis and lipid oxidation/secretion in the liver is a main characteristic of NAFLD. The excess of free fatty acids (FFA), free cholesterol (FC), and other toxic lipid metabolites cause mitochondrial dysfunction in the liver [34]. Chronic production of acetyl-CoA through oxidative processes due to the increased content of FFAs impairs ATP synthesis and increases ROS production [35]. Experimental studies have demonstrated that alteration of mitochondrial activity can be a protective mechanism to counteract the lipid overload [36,37]. In addition, FFAs indirectly affect mitochondrial function, by inducting lysosomal permeabilization and involving the activation of pro-apoptotic proteins of the Bcl-2 family [38].

In NAFLD, lysosomal dysfunction leads to reduced activity of lysosomal acid lipase (LAL), an enzyme hydrolyzing cholesterol esters and triglycerides. Clinical studies have shown a down-regulation of LAL in patients with NAFLD [39,40,41]. Serum LAL activity has been also demonstrated to be negatively correlated with the severity of NAFLD-induced liver fibrosis [42]. 

Exosomes have been identified as effective cell–cell communicators that contain a specific load from the cell of origin. They are able to transfer this load to a target or an acceptor cell, with consequent modification of the activity of the recipient cell. Abundant evidence suggests that exosomes are involved in many liver injuries, such as alcoholic and nonalcoholic fatty liver disease, liver fibrosis, cirrhosis, and hepatocellular carcinoma [43]. The exosomes are produced not only from parenchymal cells (hepatocytes), but also from non-parenchymal cells (hepatic stellate cells, endothelial, cholangiocytes, Kupfer cells, and liver endothelial cells, are directly involved in the progression and evolution of NAFLD [44].

NAFLD is characterized by changes in the levels of various fatty acids species in cell membranes. Higher levels of saturated fatty acids (SFAs) and a significant decrease of total omega-3 and omega-6 Polyunsaturated Fatty Acids (PUFAs) have been observed in different studies that have adopted a lipidomic approach for the study of NAFLD [45,46,47]. Lipidomic studies in patients with liver steatosis are substantially increased in the last decade, as well as all the studies suggesting that gut microbiota and its metabolites are relevant in NAFLD onset [48,49]. Currently, qualitative and quantitative changes in gut microbiome composition have been also associated with NAFLD stages [50].

It has also been suggested that NAFLD can potentially progress into non-alcoholic steatohepatitis (NASH), later into cirrhosis and hepatocellular carcinoma (HCC) [51]. Moreover, it has been suggested that NAFLD is a risk factor also for extrahepatic cancers, as colorectal cancer [52]. One of the potential mechanisms responsible for oncogenesis is certainly the excess of advanced glycation end products and other factors that can elicit cellular oxidative stress [52]. A retrospective study found that the incidence of HCC, as well as the non-liver mortality, were increased in patients with advanced liver fibrosis [53]. There is evidence that severe NAFLD is associated with an elevated risk of colorectal adenoma/colorectal cancer respect to mild or moderate NAFLD [54]. 

In light of this evidence, all the intervention strategies for NAFLD require a deep understanding of the metabolic defects occurring in the onset of this disease. 

The present review is aimed to focus the attention on cellular and metabolic alterations associated with NAFLD that can be helpful to identify new noninvasive biomarkers. Moreover, alternative therapeutic approaches for NAFLD, based on dietary and lifestyle modification, are encouraged.

## 2. NAFLD and Lipid Biomarkers

Liver injury is strongly associated with an increased influx of free fatty acids into hepatocytes [28], leading to alterations of cellular glucose metabolism, to a condition of insulin resistance and diabetes. Dyslipidemia associated with NAFLD is characterized by the imbalance in triglycerides metabolism, including uptake, clearance, and secretion through VLDL [55]. Dyslipidemia also consists in alteration of serum levels of specialized lipoproteins able to transport the lipids from the gut to the liver and between the liver and peripheral tissues [56,57]. In particular, high levels of circulating low density lipoproteins (LDL) have been observed in patients with liver steatosis [56,57,58,59]. 

Lipid metabolism is an active contributor to NAFLD pathophysiology so that the main lipogenic enzymes pathway result is upregulated in this pathology. Fatty acid synthase (FAS), a key enzyme in de novo lipogenesis, and Lipoprotein lipase (LPL), the rate-limiting enzyme for the hydrolysis of core triglycerides in chylomicrons and VLDL, have been detected at elevated levels in serum from patients with steatosis [58,59]. The over-expression of FAS was also associated with the degree of liver injury, suggesting that two enzymes, FAS and LPL, can be considered valid biomarkers of liver steatosis. 

Interestingly, loss of hepatic lipases function resulted in an increased liver steatosis in mice and LPL being expressed in non-parenchymal cells in the liver, is certainly involved in hepatic fibrogenesis [60].

The prevalence of NAFLD is associated with the prevalence of obesity so that a contributing factor to adverse clinical outcomes is the excess of body weight [61]. Free fatty acids released from hypertrophic adipocytes, in particular from visceral adipose tissue, lead to hyperthriglyceridemia with a consequent lipids dysfunction [62,63]. Adipose tissue expansion can result in reduced oxygen supply to the tissue, as well as to a higher production of inflammatory cytokines [64]. It has been also suggested that adipose tissue macrophages can activate Toll-like receptor 4 signaling and change their phenotype in the pro-inflammatory macrophages able to produce tumor necrosis factors-α (TNF-α) and interleukin-6 (IL-6) [64].

Additionally, recent studies demonstrated that a reduced oxygen supply to the cell, named hypoxia, is an important factor that worsens the toxic effects of lipids within hepatocytes [41,65]. Hypoxia signaling may act as a link between NAFLD and more severe forms of liver injury, as NASH [66].

Lipid accumulation in the liver creates a lipotoxic environmental leading also to mitochondrial dysfunction characterized by a decrease of cellular energy production responsible for the break of mitochondria and other hepatocellular structures [34].

Different studies, using animal models of NAFLD, have demonstrated an altered mitochondrial structure within hepatocytes, notably larger mitochondria, rounded cristae, loss of typical granules, and even mitochondrial DNA modification [67,68].

In the liver cells of NAFLD patients, a decreased mitochondrial membrane potential (MMP) has been observed [69]. This defect leads to decreased ATP-production and elevated mitochondrial production of oxygen species, responsible of oxidative stress and cell apoptosis [70]. In this regard, NAFLD is also characterized by an increased lipid peroxidation which results in altered circulating levels of oxidative stress markers [71,72]. Oxidative stress has been recognized as a central mechanism contributing to liver damage, accelerating the transition from simple steatosis to NASH [73]. A significant increase of plasma Thiobarbituric Acid Reactive Substances (TBARS) levels has been detected in NAFLD patients compared to control health subjects [74].

Laboratory approach to study NAFLD-associated changes of individual lipid species or markers of inflammation should be planned in order to identify valid disease biomarkers. About this, the analysis of circulating LPL and FAS levels, as well as the analysis of a serum inflammatory and oxidative stress profile, in particular the evaluation of circulating levels of TBARS, are proposed as possible new lipid biomarkers for NAFLD.

## 3. NAFLD and Circulating Cytokeratins

The liver damage in patients with NAFLD is accompanied by cell death that occurs primarily by apoptosis [75], which might be one of the drivers involved in the progression of NAFLD ranging from simple hepatic steatosis to NASH, with and without fibrosis, and finally to cirrhosis or hepatocarcinoma.

During hepatocytes apoptosis, caspases are activated, and they cleave a wide number of substrates, including the intermediate filament keratin 18 (K18). Then, during liver injury, both intact K18 and its fragments (CK18) can be detected in the blood. 

Expression of circulating CK-18 protein has been detected in HCC due to an instability and disorganization of the cytoskeleton system, associated with an abnormal modulation of the intermediate filaments such as cytokeratins [76]. An unstable cytoskeleton may play a role in tumor transformation and progression, local invasion, and distant metastasis [76,77].

Recent studies suggest the use of K18 and cK18 as biomarkers in the diagnosis of NAFLD and NASH [75,78,79], as well as in the monitoring of patients, in response to treatment [80,81]. The first study demonstrating the importance of caspase-generated fragments in patients with NAFLD was published in 2006 [82]. This study demonstrated that CK18 fragment levels not only were higher in subjects with NAFLD respect to controls, but they were also correlated to grade of fibrosis and inflammation stage. 

CK18-M30 and CK18-M65 are antigens of the same protein, in particular M30 measures the caspase-cleaved CK-18 revealed during apoptosis, while M65 measures both caspase-cleaved and intact CK-18, which is released from cells undergoing necrosis [83]. In patients with NAFLD, a significant positive association was detected between serum levels of CK-18 and serum Alanine aminotransferase (ALT), suggesting that CK-18 can be a convenient biomarker for making the diagnosis of NAFLD [84]. Similarly, throughout the hepatocyte apoptosis, the cleaved pieces of CK-18 can be used to differentiate the stages of liver disease [84,85].

**Figure 1:**
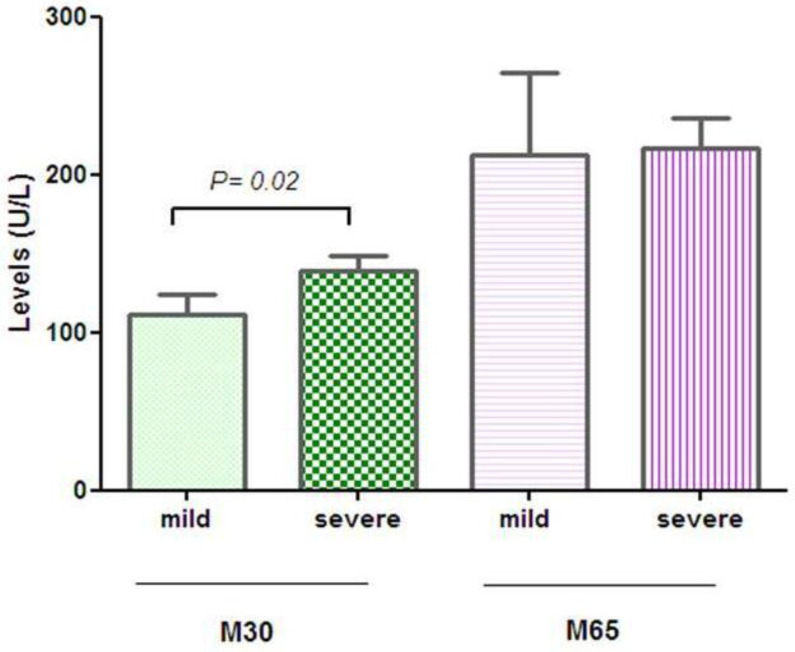
Serum levels of CK18-M30 and -M65 fragments.

However, the data are conflicting due to recent studies that have shown low sensitivity and poor diagnostic performance of CK18 tests [86,87]. Our preliminary data on the evaluation of circulating levels of CK18-M30 fragments and CK18-M65 with ELISA Kit-PEVIVA (DiaPharma) have demonstrated that the subjects with severe steatosis had significantly higher plasma levels of CK18-M30 fragments compared to subjects with mild steatosis (Mann–Whitney Test). No statistically significant difference was observed in CK18-M65 fragment levels between two groups of NAFLD patients studied (Figure 1). The CK18-M30 is specifically indicative of apoptosis, whereas the CK18-M65 is indicative of total cell death (apoptosis and necrosis).

These findings suggest that analysis of the circulating levels of CK18-M30 fragments may be helpful for the identification of the subjects with a higher degree of liver steatosis.

## 4. NAFLD and Circulating Exosomes

**Figure 2:**
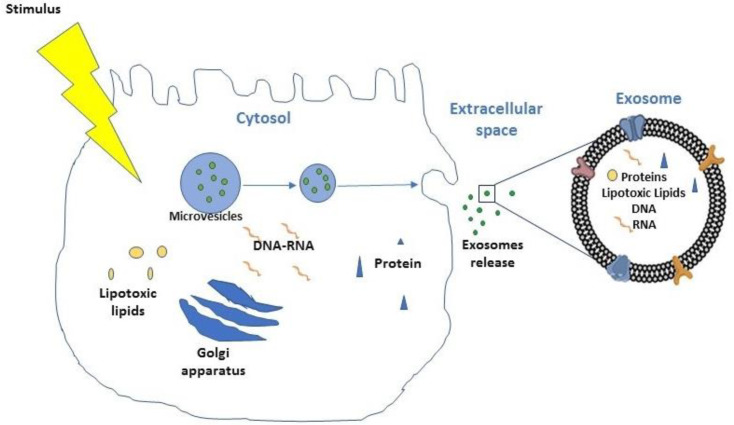
Genesis of exosome.

NAFLD progression is characterized by the accumulation of toxic lipids, such as saturated free fatty acids (SFA), ceramide, free cholesterol, or sphingolipid in hepatocytes [38,43]. The hepatocytes that receive the lipotoxic lipids release large quantities of exosomes (Figure 2), that contribute to the processes involved in the NAFLD pathogenesis [88,89], including inflammation, angiogenesis, and fibrosis [90,91,92].

Different authors have reported that mixed lineage kinase 3 (MLK3) mediates the release of hepatocyte-exosomes that carry chemokine (C-X-C motif) ligand 10 (CXCL10), a macrophage chemo-attractant, with consequent activation of hepatic macrophages (Kupffer cells) during NASH and NAFLD progression [93]. Recently, extracellular vesicles (EVs) have been considered an experimental new component of blood stream for the diagnosis of NAFLD. Extracellular vesicles, in particular the fraction named exosomes, transport different molecules including cytoplasmic proteins, lipids, DNA, mitochondrial DNA (mtDNA), transfer RNA (tRNA), messenger RNA (mRNA), microRNA (miRNA), ribosomal RNA (rRNA), noncoding RNAs (ncRNAs), and lipotoxic lipids. They consent the interaction between neighbor or distant target cells by the release in the bloodstream [94,95]. The exosomes are the smallest vesicles (30–150 nm) and are formed from the intraluminal vesicles within multivesicular bodies (MVBs), and released out of the cells after the fusion with the plasma membrane [96]. The mechanisms implicated in NAFLD progression, all related to metabolic syndrome–associated lipotoxicity, trigger the secretion of exosomes by affected hepatocytes [97]. The exosomes secreted by derived alcohol-treated hepatocytes transfer miR-122 to monocytes, with a consequent increase of sensitization to lipopolysaccharides and augmented alcohol-related inflammatory response [98]. Different proteins vehiculated from exosomes are normally upregulated or downregulated in the hepatic cells during the NAFLD evolution, such as Frizzled (FZD) proteins naturally present on the cell membrane as a Wnt receptors. In the case of hepatocyte, the protein involved is FZD-7. The FZD protein receptors are a family member of G protein-coupled receptors (GPCRs) having a substantial role as “cancer drivers” [99]. In hepatocellular carcinoma (HCC), the Wnt signaling pathway is frequently activated, and it is associated with more aggressive tumor phenotype [100]. In particular, in NAFLD progression, the FZD7 upregulation has been postulated to be an early event in liver injury [101]. During the evolution in liver fibrosis, the Hepatic stellate cells (HSCs) also play a crucial role [102] through the activation and expression of several fibrosis markers such as transforming growth factor β (TGF-β), tissue inhibitor of metalloproteinases 1 and 2 (TIMP-1 and TIMP-2), and matrix metalloproteinase-2 [103]. Young-Sun Lee et al. demonstrated that the exosomes derived from palmitic acid treated hepatocytes caused an increase in the expression levels of fibrotic genes in HSCs, with the clear demonstration that exosomes play an important role in the crosstalk between hepatocytes and HSCs in the progression from simple steatosis to NASH [104]. These changes were associated with the exosomes cargo miR-128-3p, which regulates different proteins involved in HSCs activation and liver fibrosis, one of all the peroxisome proliferator-activated receptor γ (PPAR-γ) mediators in the maintenance of a quiescent HSCs phenotype in normal liver [105].

A recent in vivo study highlights that the proliferation of primary murine hepatocytes was enhanced by self-derived exosomes [106]. Koeck et al. [107], on the contrary, demonstrated that the external exosomes derived from visceral adipose tissue were involved in the progression of NAFLD, through the dysregulation of the TGF-β pathway in hepatocytes. 

For all these aspects, we believe that, in the future, it will be useful to consider exosomes and their cargo as novel biomarkers for liver diseases.

## 5. NAFLD and Lipidomic Analysis

The fatty acids composition of cell membranes can be considered an efficacious biomarker for metabolic diseases, as obesity, diabetes mellitus, and different types of cancer [108,109,110]. The quality and the quantity of accumulated lipids in the cell membranes provide exhaustive information on the health status of the single cell, as well as of the entire organism. Therefore, the lipidomic analysis offers the opportunity to detect specific fatty acid profiles, typical of a metabolic disorder.

The lipidomic analysis on the erythrocyte membrane as “reporter” for liver injury is well recognized [1,111]. The morphology, permeability, and fluidity, as well as the membrane fatty acids composition of red cell blood and hepatocytes, are very similar [1]. This similarity allows to investigate fatty acids quality and quantity of erythrocytes membrane in order to assess alteration of lipid metabolism occurring in the liver. 

NAFLD has been often associated with an increase of saturated fatty acids and the reduction of PUFAs, especially omega-3 series, in cell membranes [43,112,113]. Previously, we demonstrated that the subjects with severe NAFLD showed a significant decrease of stearic acid/oleic acid ratio (saturation index, SI) compared to controls [114]. Low levels of SI in erythrocyte cell membranes were inversely associated with the degree of liver damage. Moreover, in the same patients, severe NAFLD was also associated with higher levels of elongase 5 enzymatic activity, estimated as vaccenic acid to palmitoleic acid ratio. This finding is in agreement with other studies showing that elevated circulating levels of cis-vaccenic acid are associated with an increased risk of coronary heart disease and stroke [115,116]. 

The lipidomic approach is relevant in patients with NAFLD, overall due to its potential in diagnosis and staging. In particular, the evaluation of SI in red blood cell membranes allows to gain novel insights in the NAFLD biomarkers discovery.

The interaction between the quality of fatty acids in the membranes and cell homeostasis is mainly mediated by omega-3/omega-6 Polyunsaturated Fatty Acids (PUFAs) ratio. PUFAs are able to modulate the inflammatory processes, likely omega-6 fatty acids have a pro-inflammatory and pro-thrombotic action, while omega-3 PUFAs are known to exert anti-oxidant and anti-inflammatory effects [117,118]. Arachidonic acid (AA) and eicosapentaenoic acid (EPA) are considered the active biological forms of omega-6 and omega-3 PUFAs, respectively, and their ratio is considered a specific index of cell inflammation [119]. An unbalanced AA/EPA ratio in favor of AA correlates with different metabolic disorders, including NAFLD [120,121,122]. Recently, a nutritional clinical trial, enrolling subjects with NAFLD, demonstrated that the combined effect of diet and physical activity reduced the AA/EPA ratio value, improving the score of steatosis [123].

AA/EPA ratio has been demonstrated to be more sensitive and accurate than the omega-6/omega-3 PUFAs in the evaluation of cell membrane inflammation status [119]. There is a direct relationship between AA levels and chronic inflammation, as well as EPA has been demonstrated to exert anti-inflammatory effects and to prevent oxidative stress within the cell membrane. In this regard, previously, we observed a lower percentage of total omega-3 PUFAs in erythrocyte membranes of patients with colorectal cancer when compared to subjects with no malignant disease [124]. Moreover, elevated AA/EPA ratio has been considered an inflammatory biomarker in tumor tissue of metastatic colorectal cancer patients [125].

**Figure 3:**
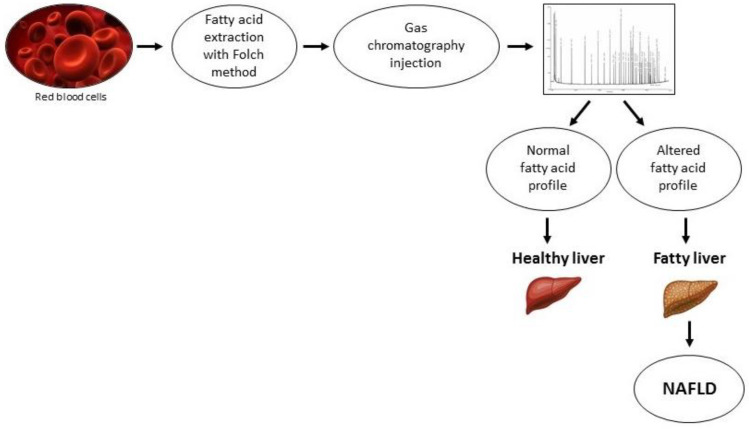
Lipidomic analysis in nonalcoholic fatty liver disease (NAFLD).

Without a doubt, abnormalities in the AA and EPA levels have critical effects on tissue inflammation status. The oxidative stress, inflammation, and dyslipidemia are the main hallmarks of NAFLD, and then it is likely that the measurement of circulating fatty acids can be used as predictive and diagnostic tools in metabolic disease as NAFLD. The rapid progress of lipidomics technology allows us to expand the identification of specific products of lipid metabolism pathways involved in the pathogenesis of NAFLD. Figure 3 shows a schematic representation of the main steps of lipidomic analysis, which is a valid tool for NAFLD diagnosis. In particular, an altered fatty acid profile observed in membranes of red blood cells might be indicative of a liver injury. 

## 6. NAFLD and Microbiota

Gut microbiota dysregulation has many implications in the development and progression of NAFLD [48]. Gut dysbiosis has been linked to the pathogenesis of NAFLD and to the progression in more severe forms of the disease [126]. Bacterial overgrowth may adversely impact metabolic pathways, as well as the immune response, favoring dysmetabolism-related diseases, including NAFLD [127]. 

The gut function is considered a crucial factor in the pathogenesis of NAFLD, and potential therapeutic strategies aimed to manage NAFLD by modulating microbiota and gut mucosa function have been tested in both adult and pediatric populations. Modification of intestinal barrier integrity has been observed in patients with NAFLD [128]. The increased gut permeability leads to the translocation of bacteria and their metabolites into circulation, contributing to the increase of circulating toxins and to the establishment of a chronic inflammatory state, a peculiar feature of many metabolic diseases [128]. Dysbiosis might also directly affect adipose tissue, influencing levels of adipokines, pro-inflammatory and anti-inflammatory cytokines, as well as fatty oxidation, all processes which have important downstream effects in the liver [129,130].

Several lines of evidence suggest that dysbiosis might increase intestinal production of ethanol due to specific bacteria [131]. Therefore, ethanol produced in the gut contributes to liver injury, exerting direct toxic effects in hepatic tissue. Fatty liver damage can be considered a response to a wide range of insults derivating by different initiating factors. 

## 7. NAFLD and Lifestyle Interventions

The modern lifestyle associated with the use of processed food and lack of physical exercise play an important role in NAFLD development [132]. The oxidative stress induced by an unhealthy lifestyle seems to be one of the causes of liver injury [133], as well as the excessive hepatic lipid accumulation.

At the cellular level, the impact of toxic lipids is detrimental for several cellular districts, including cell membrane. Changes in membrane fatty acids composition cause alteration of physical properties of the cell membrane, increasing their fluidity, and, consequently, the cell is more susceptible to lysis [134]. Therefore, any attempt to counteract cellular lipotoxicity and cell oxidative stress is considered as a therapeutic strategy for NAFLD. 

Lipidomic studies have demonstrated that hepatic fat accumulation is influenced by dietary habits and lifestyle [1,114,123]. Controlled diet aimed to reduce the assumption of linoleic and arachidonic acid levels may lead to better control of inflammatory and vasoactive metabolites associated with NAFLD and with its progression to more severe forms [46,47].

Functional alterations of lipid peroxidation determine the release of AA from phospholipids which in turn sustains lipotoxicity and liver cell damage. In this regard, diet is considered as the major factor influencing fatty acid composition in the liver.

Different clinical studies have shown that diets enriched of fruits and vegetables are able to reduce the cellular mediators of inflammation, alleviating NAFLD and its related comorbidities [135,136]. Although mostly demonstrated in animal models, flavonoids seem to have protective effects in NAFLD prevention, development, and complications [137]. It has been suggested that flavonoids may decrease body weight and fat accumulation in the liver, partly due to an increased fatty acid β-oxidation and suppressing lipogenesis [137]. The greater effects in reducing NAFLD score were achieved with dietary strategies showing high adherence to the Mediterranean diet [135]. The nutritional intervention based on the Mediterranean dietary pattern and aerobic physical activity results to be very efficacious in the improvement of NAFLD [123]. Particularly, the use of different diets, alone and in combination with two physical activity programs, demonstrated that the combination of aerobic physical activity and a Low Glycemic Index Mediterranean Diet (LGIMD) was more efficacious in reducing AA/EPA levels in erythrocytes membranes of patients with NAFLD. The reduction of AA/EPA ratio value, after 45 days of treatment, was associated with the improvement of the score of steatosis in the same subjects [123].

Adherence to a healthy diet has been reported to be essential for the primary prevention of liver steatosis [4,72]. Several studies suggested that the physical exercise might interfere with the synthesis of fatty acids, exerting a beneficial effect on liver metabolism. Weight loss is often associated with a reduction of visceral adiposity improving NAFLD patient outcomes.

Currently, lifestyle modifications remain the main treatment for NAFLD. However, it is clear that the general recommendations for diet and lifestyle changes must be always personalized in order to establish a valid therapeutic option.

Our recent study [138] showed that some food groups components were associated with a lower or a higher risk of developing severe NAFLD, and that, within the same food group, some components had a protective or promoter action. In addition, we demonstrated that both the way food is produced and the way animals are bred could play a role in rendering these foods promoters of the risk of worsening NAFLD. 

Correct dietary habits are important for the regulation of cell membrane in the liver, considered an organ leader in maintaining lipid balance. The combination of diet and physical activity has been considered effective in the prevention of NAFLD [139], so that, clinical studies have demonstrated that both the aerobic and a resistance exercise program physical activity, alone or combined, are capable of reducing NAFLD scores [140,141].

There is evidence of reversal of liver fibrosis with weight loss, so that modification of diet, physical activity, and weight loss are advocated for patients with NAFLD [142]. Regarding diet, a clinical study reported that a carbohydrate-restricted diet may be more effective in the reduction of hepatic steatosis than a low fat-diet, probably, due to a reduced content of fructose [143]. Excessive use of fructose has been demonstrated to increase plasma triglycerides and de novo lipogenesis in the liver [144].

Furthermore, since alteration of gut microflora composition contributes to liver damage, therapeutic approaches to modulate dysbiosis have been proposed as treatment of NAFLD [48]. The study of the microbiome, its functions, and interactions with lifestyle factors will provide further insights into mechanisms underlying steatosis and can offer new opportunities for therapy. Increasing efforts have been addressed to investigate the ability of probiotics to reverse gut dysbiosis in NAFLD patients [48,145]. Probiotic bacteria are capable of stimulating specific and nonspecific defense mechanisms in humans, so that, their consumption is recommended when the immunity of an organism weakens and in a condition of inflammation or infection [146]. 

It has been widely demonstrated that probiotics exert beneficial effects on dysbiosis NAFLD-related, likely for their antimicrobial properties, enhancement of mucosal barrier integrity, and immune modulation [49]. Additionally, different experimental evidence has suggested that lifestyle factors, as quality of sleep, exercise, or the use of probiotics, prebiotics, and symbiotics can have specific gut and liver effects that influence the progression of NAFLD [147,148].

## 8. Conclusions

Table 1 summarized the possible biomarkers for NAFLD proposed in this study, including their location and biological effect.

This review wanted to emphasize the beneficial effects of dietary interventions on NAFLD, as a therapeutic strategy to introduce in clinical practice and to suggest the analysis of lipidomic profile, as well as the study of exosomes proteins cargo as fundamental tools in diagnosis and follow-up of patients with NAFLD. The key message of this review is the idea that improving defenses against liver fat accumulation, through modulation of lifestyle and diet, offers a promising means to manage or treat patients with NAFLD. However, considering that NAFLD involves numerous genetic, epigenetic, and metabolic factors, this review reports some of the aspects involved in the development of the disease, limiting the discussion especially on the metabolic alterations occurring in NAFLD pathogenesis. 

In conclusion, in the future, undoubtedly new determinants of NAFLD will be identified, and the accurate assessment of NAFLD-associated risk factors will be useful to target individualized appropriate treatments. 

## Figures and Tables

**Table 1:** Possible biomarkers and their biological effect in NAFLD.

Biomarkers	Main Location	Effect
LPL	Serum	Increases the hydrolysis of triglycerides in chylomicrons and VLDL
FAS	Serum	Increases fatty acids synthesis
TBARS	Serum	Increases cell oxidative stress
Cytokeratins	Serum	Increases cell death
Exosome	Plasma	Increases cell lipotoxic lipids
Fatty acids profile	Cell membrane	Alteration of cell membrane fluidity
Microbiota	Gut	Dysbiosis and increased gut permeability

LPL: Lipoprotein lipase; VLDL: Very Low Density Lipoprotein; FAS: Fatty acid synthase; TBARS: Thiobarbituric Acid Reactive Substances.

## Data Availability

Data are available from corresponding author upon reasonable request.
